# Prevalence of Human Papillomavirus (HPV)-16 in Different Dental Infections in the Lebanese Population

**DOI:** 10.7759/cureus.38809

**Published:** 2023-05-09

**Authors:** Wael Khalil, Ferdos Alaa El Din, Marwa Jaffal, Abd El Hadi Kanj, Ali Nabbouh, Mazen Kurban, Elias A Rahal, Ghassan M Matar

**Affiliations:** 1 Oral and Maxillofacial Surgery Department, Lebanese University, Beirut, LBN; 2 Department of Microbiology, College of Dentistry, Al-Ayen University, Thi-Qar, IRQ; 3 Restorative and Esthetic Dentistry, Saint Joseph University, Beirut, LBN; 4 Department of Orthodontics, College of Dentistry, Al-Ayen University, Thi-Qar, IRQ; 5 Office of Graduate Studies in Biomedical Sciences, American University of Beirut, Beirut, LBN; 6 Dermatopathology, American University of Beirut Medical Center, Beirut, LBN; 7 Department of Experimental Pathology, Immunology, and Microbiology, American University of Beirut, Beirut, LBN

**Keywords:** peri-apical infection, virus, microbiology, pcr, pericoronitis, periodontitis

## Abstract

Background: Dental infections, which are the main cause of tooth loss, are known to be caused by bacteria. However, recent research suggests that other organisms, such as viruses, may also play a role. In this study, we aim to detect the presence of human papillomavirus (HPV)-16 and assess its prevalence in tissues infected with various dental infections, including aggressive and chronic periodontitis, pericoronitis, and periapical infection, as well as healthy gingival tissues, saliva, and gingival crevicular fluid, for comparison.

Methods: A cross-sectional study including 124 adult healthy patients presenting with dental infections requiring dental extractions were conducted to assess the prevalence of HPV-16 in saliva, infected, and healthy tissues using quantitative polymerase chain reaction (PCR) tests. Samples were collected and a categorical scale was used for the prevalence. Statistical analyses were performed using Chi-square for the prevalence of HPV-16.

Results: Among the HPV-16-positive PCR cases, the prevalence of HPV-16 was highest in periapical infection tissues as compared to chronic periodontitis, aggressive periodontitis, pericoronitis, and control tissues.

Conclusion: The prevalence of HPV-16 in periapical infection samples was the highest among the studied dental infection samples. Thus, a primary conclusion can be drawn about the presence of an association between HPV-16 and the occurrence of periapical infection.

## Introduction

Dental infections, or infections of dental origin, belong to the most commonly encountered infections in the oral cavity. They are considered the main cause of tooth loss as they affect the tooth-supporting tissues such as in periodontitis, or the periapical alveolar bone when it comes to periapical infection or abscess, or the hard and soft tissues surrounding an erupting third molar causing pericoronitis [1]. It has been well-established in the literature that bacteria are the main cause of these infections, but recent research suggests that other organisms, such as viruses, may also play a role [2]. Human papillomavirus (HPV)-16 which belongs to the Papillomaviridae family is one of the most common viruses that infect oral mucosa and surrounding skin [3]. This virus can also be found in the normal oral cavity flora [4]. However, there is controversy in the published reports concerning the prevalence of various viruses, particularly HPV-16, in different dental infections, which raises several questions about their presence and role in the oral cavity. In this study, we aim to detect the presence of HPV-16 and assess its prevalence in tissues infected with various dental infections, including aggressive and chronic periodontitis (AP; CP), pericoronitis (PC), and periapical infection (PI), as well as healthy gingival tissues, saliva, and gingival crevicular fluid (GCF) for comparison.

## Materials and methods

Study design

This study was designed as a cross-sectional study that included 124 adult healthy patients. The patients were recruited from a private clinic in Beirut, Lebanon, between January 2020 and January 2022 using a non-probability convenient sampling technique. The tissue or saliva specimens were collected by one clinician and all samples were evaluated by one examiner who was blinded to the disease conditions of the patients.

Ethics statement

**Figure 1:**
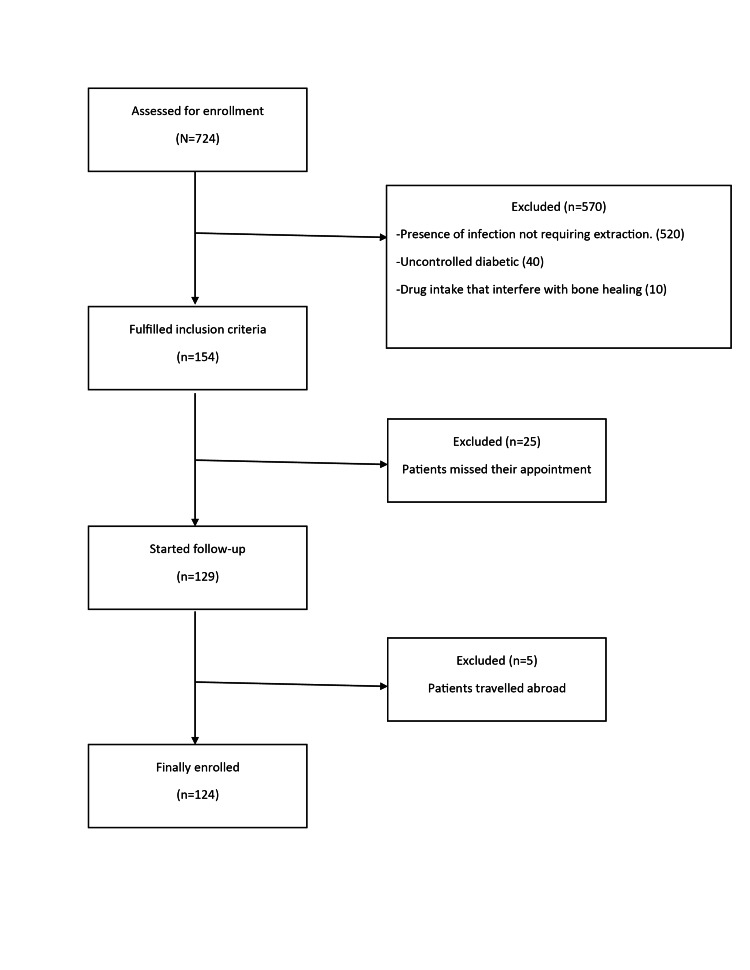
Study Design and Strengthening the Reporting of Observational Studies in Epidemiology (STROBE) Flowchart

The study was designed and conducted based on the Strengthening the Reporting of Observational Studies in Epidemiology (STROBE) and a flow diagram was prepared (Figure 1). The study was performed according to the most recent Declaration of Helsinki guidelines for clinical trials involving human subjects. Ethical approval was obtained from the Institutional Review Board at the American University of Beirut, Beirut, Lebanon (Protocol number: BIO-2019-0511). Patients fulfilling the inclusion criteria were recruited in the study and a consent form was explained verbally in detail including information on the scope and benefits of the study as well as the associated risks. A copy of the consent form is attached. The form was signed by all participants in the study. 

Inclusion criteria

Adult healthy patients, American Society of Anesthesiologists (ASA) I, who presented with dental infections that included AP, CP, PI or PC infections and requiring dental extractions were included in this study.

Exclusion criteria

Exclusion criteria included children and medically compromised patients (ASA II and III), including non-consenting patients with a history of cognitive dysfunction, pregnant or lactating women, as well as patients undergoing chemotherapy or radiotherapy.

Sample size determination

The sample size was determined based on the primary outcome, prevalence, with a 95% confidence interval assuming an alpha of 5%, an estimated prevalence of 50%, and a margin of error of 10%. 

Sample collection

Infected gingival tissues were collected from extraction sockets of teeth with either one of the studied pathologies, while healthy gingival tissue samples were collected from extraction sockets of teeth that were extracted for orthodontic or prosthetic reasons and were confirmed to be healthy.

Saliva and GCF samples were collected from some of the participants and analyzed for the presence of HPV-16. Patients were provided with a sterile container in which to spit. GCF samples were collected by inserting paper points into the gingival sulcus. Infected and healthy tissues were collected from both the socket and/or the periodontal ligament of the extracted teeth. Sample tissues were then rinsed with a sterile saline solution, and all samples were stored at -80°C for future analysis.

Outcome measures

The primary outcome was the prevalence of HPV-16, which was evaluated and reported as a ratio in saliva, GCF, healthy and infected samples.

Sampling quantification of HPV-16

DNA extraction and isolation were performed using the QIAamp® DNA mini kit (Qiagen, Gaithersburg, MD, USA), as per manufacturers' instructions, through three main steps: lysis, followed by DNA precipitation, and DNA elution. DNA purity and yield were evaluated using a nanodrop spectrophotometer (Denovix, Wilmington, DE, USA) in addition to q-PCR analysis (Numelab, Beirut, Lebanon).

Quantitative polymerase chain reaction analyses (q-PCR)

The q-PCR technique was used to determine the presence of HPV-16 in fluid and tissue samples. The q-PCR technique was conducted using a SYBR green mix in a CFX96 system (Bio-Rad, Hercules, CA, USA). Products were amplified using primers detailed in Table 1. PCR settings were as follows: a pre-cycle at 95°C for three minutes followed by 40 cycles each consisting of 95°C (denaturation) for 15 seconds, the annealing temperature for 30 seconds, and 72°C (elongation) for 30 seconds. The fluorescence threshold cycle value (Ct) was recorded for each sample. The positive control was the DNA of the pathogen, and the negative control was water. The result was analyzed using Rotor-Gene Q series software (Rotor-Gene, Sydney, Australia), in which a cycle threshold value (Ct-value) < 40 for HPV-16 gene was defined as a positive result.

**Table 1:** Quantitative polymerase chain reaction (q-PCR) primers and annealing temperatures of genes used to detect the DNA of the pathogens.

Primers	Sequence	Annealing T (◦C)
Human papillomavirus-16	F: CCCAGCTGTAATCATGCATGGAGA R: GTGTGCCCATTAACAGGTCTTCC	52

## Results

Seven hundred twenty-four patients were screened, of which 570 patients were excluded, and 154 patients included. Among the recruited patients, 124 finally participated in the study. Tissue samples were obtained from 32 CP, 38 PI, 27 PC, and 17 AP (Table 2) in addition to 79 saliva and 41 GCF samples that were collected.

**Table 2:** Distribution of infected tissues samples per age and gender

Dental infection	Total	Males	Females	Age range
Pericoronitis	27	12	15	17-35
Chronic Periodontitis	32	23	9	50-67
Aggressive Periodontitis	17	9	8	16-28
Periapical Infection	38	20	18	18-70
Saliva	79	57	22	17-70
Gingival Crevicular Fluid	41	30	11	17-70

Prevalence of HPV-16 virus

Among the HPV-16-positive PCR cases, the prevalence of HPV-16 was highest in PI (23.68%) tissues as compared to CP (15.62%), AP (7.14%), PC (5.88%), and control (9.75%) tissues. HPV-16 was detected in 3.79% of the saliva samples and no positive cases of HPV-16 were detected in any of the GCF samples (Table 3).

**Table 3:** The prevalence of HPV-16 and its confidence intervals among the control healthy tissues, infection lesions, and saliva and GCF samples S: Saliva; CP: chronic periodontitis; AP: aggressive periodontitis; PI: periapical infection; PC: pericoronitis; C: Healthy Control; GCF: Gingival Crevicular Fluid

Pathogen	Prevalence (%)		
HPV-16		Positive	Negative
	C	9.75 [4.31-18.32]	90.25 [81.68-95.69]
	CP	15.62 [5.28-32.79]	84.3 [67.21-94.72]
	PI	23.68 [11.44-40.24]	76.32 [59.76-88.56]
	AP	5.88 [0.15-28.69]	94.12 [71.31-99.85]
	PC	7.41 [0.91-24.29]	92.59 [75.71-99.09]
	S	3.79 [0.79-10.70]	96.21 [89.30-99.21]
	GCF	0	100

## Discussion

The oral cavity is a reservoir for HPV, which has been linked to the development of various diseases. In healthy individuals, HPV may be present at a low level, but it is capable of amplification in suitable environments, particularly when the host immunity deteriorates [5].

The prevalence of HPV in the normal oral cavity has been reported in several studies, with considerable variation. For example, a study of 100 young volunteers from Brazil using PCR analysis found no HPV [6], whereas a high prevalence of HPV in the normal oral cavity (81%) was found in Japanese adults [7]. In our study, we found a prevalence rate of 9.75% for HPV in the normal oral cavity, which is not directly comparable to other studies that reported approximately 25% of the participants as positive for HPV, using PCR analysis [8,9].

As the presence of HPV has been suspected to be associated with dental infections, we investigated the prevalence of HPV-16 in saliva, GCF, and tissues of common dental infections (PC, AP, CP, and PI) as compared to healthy tissues from each participant in the Lebanese population. Among the HPV-16-positive PCR cases, the prevalence of HPV-16 was highest in PI tissues (23.68%) as compared to other investigated infection tissues, healthy tissues, saliva and GCF samples with a confidence interval of this prevalence ranging between 11.44 and 40.24. As the confidence interval reflects the prevalence of this virus from our samples in the Lebanese population with a 5% error, the high value of the lower limit of this interval (11.44 significantly superior to zero) indicates that this virus has a significantly high prevalence in the Lebanese population. As for CP, the prevalence of HPV-16 was 15.7% with confidence interval ranging between 5.28 and 32.79, so this suggests a possible association between HPV-16 and CP in the Lebanese population. In the control samples, the prevalence of HPV-16 detected was 9.75% with confidence interval ranging between 4.31 and 18.32, which suggests that HPV-16 may be present in healthy tissues similarly like other viruses. On the other hand, the low prevalence of HPV-16 in AP, PC, and saliva (5.88, 7.41, 3.79 respectively), and the approximately zero lower limit of confidence interval (0.15, 0.91, 0.79 respectively) suggests that no association is found between AP and PC and HPV-16, and that this virus is not found in saliva. In addition, it should be noted that the values of prevalence obtained in AP and PC were close to those obtained for control tissues, which supports our conclusion of the absence of association between HPV-16 and AP and PC.

On the other hand, our results were non-conclusive concerning the association between HPV-16 and periodontitis, while some previously conducted studies did not find an association [10,11]. On the other hand, our findings were not in line with some other studies that were certain about the presence of this association. The controversy in the results may be due to several factors and conditions within each study, including sample size, pathogen detection technique, and sampling technique. The samples in previous studies were taken using various methods such as paper points or sterile curette insertion, GCF samples, saliva samples, gingival specimens, oral rinse, and mucosal scrapings [2,12-14]. These techniques are not specific to the infected tissues and may include both normal and pathogenic microorganisms. In contrast, the samples collected in our study were exclusively obtained from infected tissue (granulation tissue) at the infected sites. Furthermore, in many existing research models, authors either did not specify which HPV type was examined, or did not have control healthy tissue samples for comparison. In other studies, the comparison was based on saliva samples rather than tissue samples [10,11].

Besides, there are several factors that could contribute to the variability in prevalence and controversies between studies, such as demographic factors, systemic and oral health status, clinical tissue sampling, and laboratory techniques. The analysis was performed using a real-time PCR technique for assessing the presence of virus in each sample category and to compare the results between different sample categories. According to the available information, no previous studies have assessed and compared the viral presence of HPV-16 in all these dental infections. In the oral cavity, the detection rate of HPV varies considerably in published data, ranging from 0-100%, depending on the status of the disease, tissue origin, detection techniques, and other environmental risk factors such as geographic regions [6].

Although the presence of HPV-16 has been explored in AP, CP, and PI, the literature is scarce, particularly in PI studies [15-18]. Among all dental infections, HPV-16 showed significant differences in prevalence, particularly in PI (23.68%). The association between HPV-16 and PI reported in this study was in concordance with published studies [17,18]. To our knowledge, we were the first to investigate the association between this virus and PC. However, our results showed no significant association [19]. Similarly, our study did not find any evidence supporting an association between HPV-16 and AP or CP, although previous studies remain controversial [20,21].

It is important to point out that the quantity of HPV-16 in both fluids, saliva, and GCF, was significantly lower than all other healthy and infected tissue samples (3.79 and 5% respectively). This could be plausibly attributed to the fact that HPV-16 is an intracellular micro-organism [22] and has a specific tropism toward squamous epithelial cells [23]. Furthermore, saliva is in direct contact with the periodontal pocket in the case of AP and CP and the operculum in PC, unlike in PI where the infection is in the bone and not in direct communication with the oral cavity.

Moreover, multiple HPV genotypes may be present in dental infections. The most commonly identified genotypes are HPV-16 and HPV-18, with HPV-16 being more prevalent in the normal oral cavity than HPV-18 [4]. As viral replication often precedes the development of virus-associated pathology, identifying HPV in individual ‘at risk’ for HPV-associated diseases may be a useful prognostic indicator.

## Conclusions

In summary, our study found that the prevalence of HPV-16 was higher in tissue samples than in fluid samples such as saliva and GCF. Furthermore, infected tissue samples, and more specifically PI samples, showed a significantly higher prevalence of HPV-16. Therefore, a primary conclusion can be drawn about the presence of an association between HPV-16 and PI. This association may suggest that patients with dental infections may require special care regarding cross-contamination and their high risk of oral carcinoma due to their elevated chance of having an underlying HPV-16 infection. However, further studies and investigations are necessary to confirm this hypothesis.
